# Case of *Pneumonia*, Where the Extent to Which Blood-Letting May Be Successfully Carried Is Fully Exemplified

**Published:** 1817-12

**Authors:** 

**Affiliations:** Assistant Surgeon


*Case of* PNEUMONIA, *where the Extent to which Blood-letting may be successfully carried is fully exemplified.* By Assistant Surgeon SIMPSON, 56th Regiment.

PRIVATE George Sparr, a bugle player in the 56th regiment of foot, 23 years of age, and of a plethoric habit, was, on the 8th of May, 1817, admitted into the regimental hospital, Hilsea. He complained of violent pain in the chest, but most particularly in the right side. The pulse was full, hard, and bounding; the tongue covered with a light brown fur; the skin hot and dry; the face deeply flushed; the eyes turgid, and blood shot; thirst extreme; respiration difficult, hurried, and oppressed; excruciating pain on attempting a full inspiration; a hard cough; expectoration, pure blood. Says he was first attacked on the morning of the 7th.

Brachio dextro detrahatur sanguis ad deliquium.

Postea sumat sodæ sulphatis unciam. Cibar. liquid.

*Hora sexta vespertina.*

Syncope occurred after 24 ounces were taken; no buff. The pain is somewhat abated; but the pulse is again full and hard, and the face flushed; thirst unabated; several stools.

Repetatur venæsectio ad deliquium.—℞. Antimonii tartarisati grana quatuor; aquæ fontanæ ℔jß. Solve, dein adde magnesiæ sulphatis unciam: solve iterum. Sumat unciam quaque hora.

9th. Fainted after losing 24 ounces; still no buff. The pulse is full, hard, and bounding, and every symptom remaining in full force. Expectoration bloody.

Repetatur venæsectio; pergat in usu solutionis; sterno imponatur emplastrum lyttæ.

*Hora sexta vespertina.*

The pulse became imperceptible, after losing 10 ounces, and the vein was closed. Felt but little relief from the operation. The pulse has again become very full and hard; face flushed; skin parched; respiration laborious and rapid; urine high coloured and scanty; the expectoration is still bloody.

Repetatur venæsectio prout vires, et pergat in usu solutionis.

10th. Fainted after losing 14 ounces; no buff. Had a good night, as all the symptoms were much lessened in violence. The pulse is calm; respiration tolerably easy; bowels loose; no expectoration of blood; cough easier.

Pergat in usu solutionis.

*Hora sexta vespertina.*

Has been very easy all day; thinks he will sleep well.—Interim quiescat.

11th. After a quiet night, the pulse has again risen, and every symptom returned with redoubled violence. Pain of side extreme; thirst insatiable; respiration performed convulsively.

Repetatur venæsectio pro effectu.

Pergat in usu solutionis.

*Hora sexta.* Forty-two ounces were taken, which now show a thick and almost impenetrable coat of buff. Was extremely enfeebled by so large a depletion, but soon felt its good effects. Is still very low, but the pulse is soft, and the respiration and pain of side much easier. The bowels are loose.

Hora octava utatur balneo tepido.

12th. A tolerable night. The respiration is pretty free, but the pulse is hard and full, and the countenance slightly flushed. Bowels open; thirst abated; urine more natural, both in quantity and in colour. Still some pain of side.

Repetatur venæsectio sed cautè.

*Hora sexta vespertina.* Twenty-four ounces, buffy, were taken. Is now very easy.

Hora octava utatur balneo tepido.

13th. A good night. The tongue is clean and moist; the bowels are open, and the urine almost natural; but the cough is very troublesome. Pain of side rather increased; respiration hurried and oppressed; and the pulse full and hard.

Repetatur venæsectio prout vires.

*Hora sexta.* Thirty-eight ounces were taken, which now show a thin coat of buff. The pain of side is diminished, and the pulse is soft; but the cough is fully as severe as before. Expectoration mucous.

℞. Antimonii tart. granum. mucilag. g. acaciæ ℥viij. solve, dein adde tincturæ opii-scillæ āā ʒß. M. sumat cochleare parvum quandocunque tussis molesta sit.

14th. A tolerably comfortable night. The breathing at this moment is oppressed and hurried, and he cannot inspire freely without experiencing violent pain. Pulse again full and hard; cough frequent and severe. Again expectorates blood. Bowels open; tongue moist and white.

Repetatur vænesectio sed cautissime.

*Hora sexta.* Twenty-four ounces, shewing a thin inflammatory coat, were taken. Feels more easy.

Repetatur balneum.

15th. A good night. The expectoration of blood has again ceased, but the pulse is still full. The pain of chest severe, and the cough very annoying. Respiration hurried; bowels open.

Repetatur venæsectio cautissime.

*Hora sexta.* Lost twenty-four ounces, which have a deep thick coat of buff. Is very weak, but quite free from pain.—Quiescat.

16th. An easy night. The pulse is still full, but the debility is so very alarming, and the countenance so pallid, that no more depletion can be attempted with safety. Has still considerable pain of side and difficulty of respiration; cough troublesome. Expectorates a small quantity of mucus. Tongue white; bowels open.

Perg. in usu misturæ. Repetatur balneum tepidum.

*Hora octava.* As at the morning's report.

17th. A very comfortable night. The pain of side is abated; cough easier; tongue moist; bowels open. The pulse is soft and regular. Debility less.

Perg. in usu misturæ.

18th. A good night. Though the respiration is still hurried, yet he has less difficulty and pain. Pulse natural; bowels free; tongue clean and moist.

Perg. in usu misturæ.

19th. A very good night. Cannot yet inspire deeply without some degree of uneasiness, and exciting cough; but the pulse is soft and calm; the tongue clean and moist; and the skin genial. Is still pale and languid, but daily acquires strength. Expectoration easy, scanty, and mucous. Bowels rather costive.

Perg. in usu misturæ. Sumat. olei ricini drachmas sex.

21st. During the last two days he has been rapidly improving. The pulse, skin, and tongue are perfectly natural. The bowels open; no thirst; appetite for food keen. The cough diminishes. The expectoration free. No pain of side or chest; and can lay on either side with perfect ease. —Quiescat.

His health from this period improved with astonishing rapidity. On the second of June no vestige of his complaint remained, and on the 8th he was discharged cured; but with orders not to resume his duty as a bugle, till he had obtained a special permission to that effect.

Many instances of violent pneumonic inflammation have occurred in our regiment, but none I believe so remarkable as this—so violent in its nature; so rapid in its termination; and for the removal of which so much depletion was found necessary. I have, at a former period, detailed the case of private Patrick Connor, from whom 170 ounces were taken in seventy-three hours: but here we have the amazing sum of 224 ounces taken between the 8th and the 15th. The treatment was simple; but, happily, effectual: and I am rather inclined to think, that provided the nature of the disease be distinctly understood, the simpler the practice the more creditable will it be to the surgeon, and the more fortunate for the patient.

J. SIMPSON, Assist. Surg.

---

*Observations on Erysipelas Infantile.* By R. MOULSON, M. D. Physician to the Halifax General Dispensary.

